# A randomized controlled trial comparing self-referred message to family-referred message promoting men’s adherence to evidence-based guidelines on *BRCA1/2* germline genetic testing: A registered study protocol

**DOI:** 10.1371/journal.pone.0266327

**Published:** 2022-04-08

**Authors:** Serena Petrocchi, Giulia Ongaro, Mariarosaria Calvello, Irene Feroce, Bernardo Bonanni, Gabriella Pravettoni

**Affiliations:** 1 Applied Research Division for Cognitive and Psychological Science, IEO, European Institute of Oncology IRCCS, Milan, Italy; 2 Department of Oncology and Hemato-Oncology, University of Milan, Milan, Italy; 3 Division of Cancer Prevention and Genetics, IEO, European Institute of Oncology IRCCS, Milan, Italy; CNR, ITALY

## Abstract

**Background:**

This is a registered study protocol on a randomized controlled trial (RCT) testing an intervention aimed to improve men’s adherence to evidence-based guidelines on BRCA1/2 germline genetic testing. *BRCA1*- and *BRCA2-*associated Hereditary Breast and Ovarian Cancer Syndrome (HBOC) increases the relative and absolute risk of developing breast and ovarian cancer and, to a lesser extent, prostate and pancreatic cancer. Men face *BRCA*-related cancer risks as women do, although with a different magnitude, and they may also transmit the mutations to their children. Notwithstanding, men are under-tested compared to women and the communication is not tailored on their needs. The present RCT applies principles of the Health Action Process Approach (HAPA) in testing the psychological determinants of the men’s adherence to evidence based guidelines on BRCA1/2 germline genetic and testing the efficacy of two messages.

**Methods:**

A total of 264 participants will be involved, among the men’s relatives of women with verified germline mutations. The study entails a pre- post- evaluation with randomization of the participants in two conditions corresponding to the two messages.

**Discussion:**

The expected results provide answers related to the impact of action self-efficacy, outcome expectancy (personal or familiar), risk perception, health risk aversion, intolerance of uncertainty, perceived barriers, and coping self-efficacy on informed decision-making. Data gathered from this study may inform health care providers, policy makers, and public health managers about the communication strategy for men and about the psychological variables influencing decision-making.

**Trail registration:**

**Name of the Registry:** Clinical Trials. **Trial registration number:** NCT04683068. **Date of registration:** 16/12/2020. **URL of trial registry record:** https://www.clinicaltrials.gov/.

## Introduction

As for other pathogenic gene mutations [1–3], germline mutations in *BRCA1*- and *BRCA2* genes (BReast CAncer 1 gene and BReast CAncer 2 gene) predispose carriers to an increased susceptibility in developing breast and ovarian cancer [4] but also prostate, pancreatic cancer and melanoma [5]. *BRCA1* and *BRCA2* germline mutations are inherited in an autosomal dominant manner; this means that offspring of an individual with a *BRCA1* or *BRCA2* germline mutation have a 50% chance of inheriting the variant [6, 7], and the same probability of passing it to the progeny. *BRCA1/2* mutations have no gender distinction [8], both men and women can inherit the mutation, although it exposes them to different risks.

Men with *BRCA1/2* mutations can incur a lifetime risk of up to 6.8 per cent for breast cancer, and between 6 and 15 per cent for prostate cancer [7, 9]. These risks are particularly higher when *BRCA2* gene is involved. However, men do not receive the same attention as women [10]. In particular women can be tested to search *BRCA1* or *BRCA2* unknown germline mutations, especially when suffering from breast and/or ovarian cancer, or to search known *BRCA1/2* germline mutations previously identified in the family (i.e., cascade screening). Instead men are mainly involved in cascade screening and, rarely, they are tested for *BRCA1/2* germline mutations [11]. Very recently, the guidelines of the National Comprehensive Cancer Network [12] have suggested to consider patients with pancreatic and prostatic cancers as eligible for genetic testing for BRCA germline mutations. However, such a recommendation is not yet routine for the test proposal.

Some socio-cultural aspects have been related to the men’s attitude toward *BRCA1/2* mutations and genetic testing. For example, Pritchard [13] suggested that *BRCA 1/2* mutations are generally associated with female gender. He also pointed out that the name of the associated Hereditary Breast and Ovarian Cancer Syndrome (HBOC) creates confusion since breast and ovarian cancers are considered a ‘female matter’ by layperson [13]. Similarly, others [14, 15] have found that fear of stigmatization is one obstacle for the men’s decision-making. To date, there are no studies, applying a strong theoretical rationale, that have systematically tested which are the psychological variables influencing men’s informed decisions for genetic testing, when facing *BRCA*-related mutations. Moreover, to the best of our knowledge there are no studies testing what is the better communication strategy to inform men’s decision-making. Therefore, the present study aims to fill these gaps with an experimental study with a longitudinal component. First, applying principles of the Health Action Process Approach (HAPA), a motivational inclined theory that explains changes in behaviors, will be tested what psychological determinants influence the decision to undergo a genetic test for a germline BRCA mutations. The role of intolerance to the uncertainty and the health risk attitudes will be explored. The HAPA model has been chosen because it is an evolution of the first stadial models of health psychology [16] and it assumes that individuals pass through qualitatively different stages of psychological elaboration when they have to adopt new health behaviors. The other aim of the proposed study is to test two different narrative messages in order to understand which is the most efficient in informing men’s decision-making.

### Principles of the Health Action Process Approach (HAPA)

According to the Health Action Process Approach [17] there are several variables involved in the implementation of new health behaviors. Specifically, it is possible to identify risk perception, outcome expectancies and action self-efficacy as predisposing factors that have an impact on the informed decision; this is considered the motivation phase, in which subjects begin to form an intention. In this model, the intention to perform a specific health behavior is considered as a middle-level mediator between the variables considered in the motivation phase and those in the volition phase.

Furthermore, according to this model, volition factors involve planning that is further specified as coping and action planning and coping self-efficacy. Volition factors are considered as being influential in the subsequent phase and they are the most proximal predictors to actual behavior decision. This second phase (i.e., the volition phase) should be subdivided in pre-action phase, in which the previous mentioned coping and action planning, and coping self-efficacy take place, and action phase, ending with a behavior.

This model was tested in several health contexts [18, 19], in particular for smoking behavior [17, 20, 21], physical activity [22, 23], dietary [24, 25] and also in cancer-related screening behavior [26]. Those studies demonstrated the efficacy of the theory in explaining the initiation and maintenance of such health preventive behaviors. Together with the principles of the HAPA model, the present study aims to understand whether other psychological factors may explain men’s decision making regarding genetic testing for *BRCA1/2* germline mutations [27]. For this reason, two attitudinal factors are examined: intolerance of uncertainty and health risk attitudes.

### Intolerance of uncertainty and health risk attitudes

When people face a potential threat for their health, one key element affecting their subsequent decisions and behaviors is how much they feel certain or uncertain regarding the threat. In our case, a man who is considering the possibility to undergo a genetic test for BRCA germline mutations is facing two different types of uncertainty. The first one is the proximal uncertainty due to the result of the genetic test itself. The second one is the distal uncertainty depending on the risk to develop a cancer if a germline mutation would be found. The intention to undergo a genetic test for a germline mutation may be, therefore, determined by the way in which the individual manages the uncertainty due to the discovery of a possible negative result (i.e., the presence of a mutation) and to the future consequences of the germline mutation [28].

Intolerance of Uncertainty (IU) is a trait characteristic of individuals who are not able to tolerate the aversive reactions triggered by a perceived lack of sufficient information or by an issue that can have more than one solution [29]. Individuals with low tolerance for uncertainty tend to perceive the threat as a source of discomfort and to react negatively to it [30]. Some studies investigated the relationship between intolerance of uncertainty (IU) and the attitude to undergo health monitoring, in particular cancer-related screening [31, 32]. In particular, Tan and colleagues showed that intolerance of uncertainty may function as an important determinant of anxiety among men pursuing active surveillance for prostate cancer [31]. One qualitative study in lung cancer screening decision-making showed that some participants sought to decrease uncertainty through lung cancer screening and, if needed, with additional testing; others declined the screening in order to avoid the uncertainty associated with undefined results [33]. Indeed, intolerance of uncertainty consists of two dimensions: the first one, the desire for predictability, is an active strategy to manage uncertainty that is perceived as intolerable and leads to search for as much information as possible about the threat to restore a balance. Rosen and colleagues [34] showed that high levels of IU were associated with an increase in health monitoring and screening; other studies suggested that searching for threat-related information may be driven by the desire to reduce uncertainty [35, 36]. The second one, called uncertainty paralysis, is configured as an avoidance strategy, and leads to inability to act because of the uncertainty [29].

Another construct related to the previous one, at least in the health framework, is the attitude toward health risk. In order to contrast the perception of significant uncertainty, people choose responses and act in a certain way, and this is defined as the personal attitude toward the health risk. In fact, people differ in their attitude towards health risks, and this affects decision making regarding preventive behaviors (e.g., screening, physical activity), and risky behaviors (e.g., surgery) [37]. Taken together all this evidence suggests that IU and health risk attitudes may play a role when a man is making a decision to undergo a genetic test to detect BRCA germline mutation because there are similar mutations in the family. Since men with *BRCA*-related cancer risks deal with probabilistic and complex information [38, 39], both of these attitudinal factors can have an effect on the implementation of health behaviors and therefore can influence the informed decision to undergo cascade screening for *BRCA1/2* mutations.

### How to inform men’s decision making for *BRCA1/2* germline mutations genetic testing

The other aim is to investigate how to inform men’s decision-making. Low levels of men’s knowledge regarding *BRCA1/2* germline mutations is found as one of the most important problems in this field [40]. Therefore, one main research achievement may be to find a right way to correctly inform them. The present study aims to test two different messages tailored on men’s specific needs and to understand what is the best strategy to inform them. Although narrative approaches showed efficacy in promoting health behavioral intentions [41], and in increasing adherence to cancer screening [42] in particular, its utility for improving men’s *BRCA* cascade screening remains unexplored. The present research intends to explore whether narrative messages can be effective in informing men’s decision-making.

In particular, two features are fundamental in narrative strategies context: the narrative perspective and the framing. The first one is a fundamental story feature and changes how information is delivered to the audience; researchers identified that first-person narrative messages are able to increase self-identity and to promote the assimilation of the theme better than third-person narratives [43]. Therefore, the present study chooses to create narrative messages with a first-person feature. Regarding the framing, messages can either emphasize negative consequences (losses) or positive outcomes (gains) of a given action. Prospect theory [44] posits that, in general, people are more likely to take risks when presented with a loss-framed message, and the contrary for a gain-framed message. But regarding disease prevention behaviors, like smoking cessation [45] and skin cancer prevention [46], researchers have suggested an advantage, albeit small, of a gain frame over a loss frame [47]. Therefore, the present study chooses to create narrative messages oriented to a gain frame.

Furthermore, applying the Uncertainty Management Theory [48], a social psychological approach to uncertainty, Rauscher et al. [14] investigated how men with increased *BRCA*-related cancer risk approach the individual and familial uncertainty related to that pathogenic variant. Those qualitative results showed that men’s primary concern when managing *BRCA*-related cancer risks is the aversive consequences of the discovery of a germline mutation for their family [49]. Their focus on familial uncertainty management is maybe due to the difficulties encountered in the management of their own risk due to the lack of information and management options. In the end, the authors suggested that genetic counseling would benefit a family focus. Also, Hallowell and colleagues [50] highlighted the role of family members (mother, partner, or children) in men’s decision-making about BRCA testing. In particular, they showed how men’s decision to have genetic testing was influenced by the obligations perceived from family members, primarily their children.

Based on these premises, this study protocol proposes to test the effectiveness of two first-person gain-framed messages, one narrating a self-referred story and the other a family-referred story. The effectiveness will be measured in terms of one or both messages ability to predict the intention to undergo the genetic screening.

### Hypotheses

**Fig 1:**
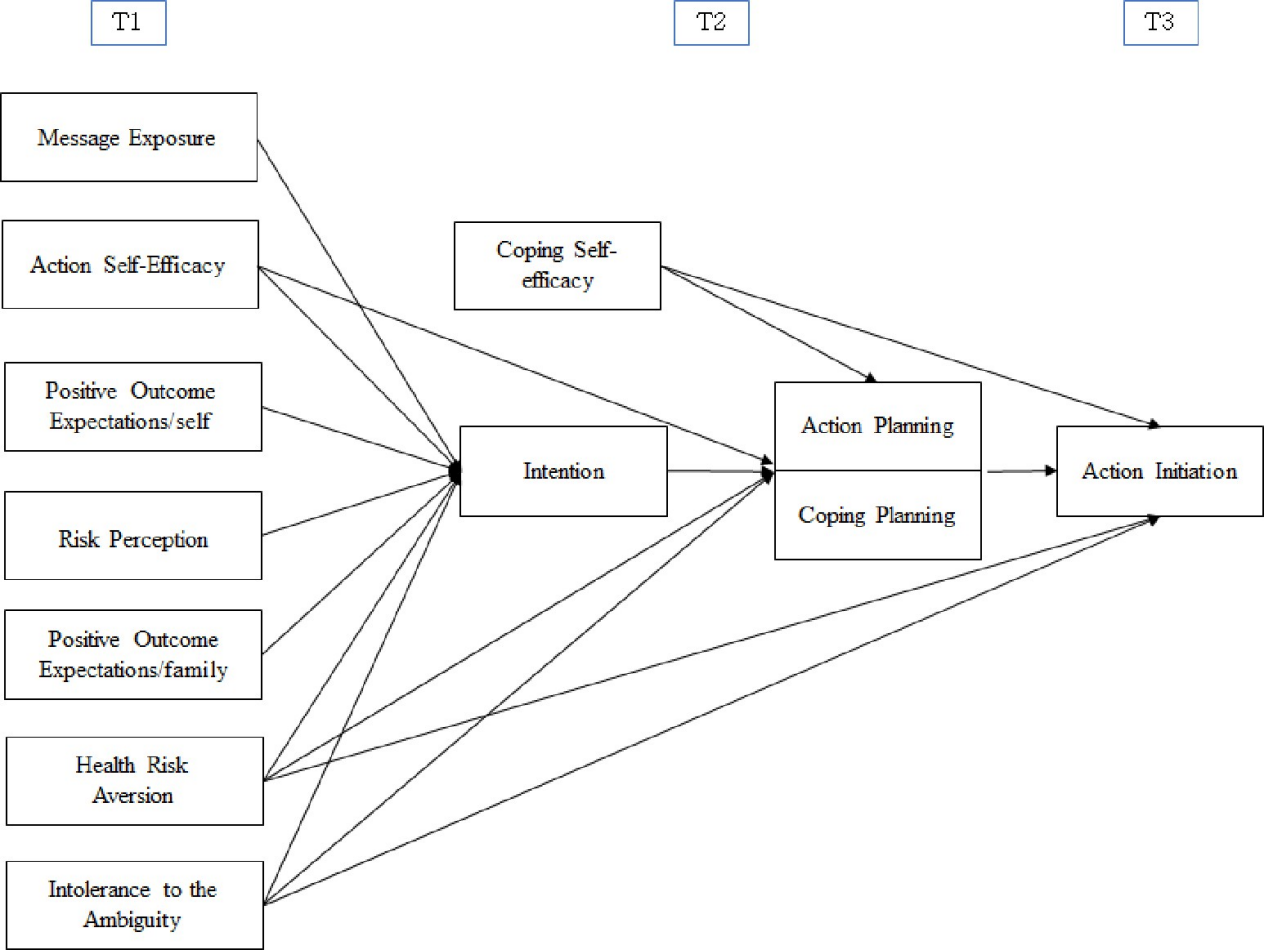
Expected relations between variables. T1, T2, T3 indicate the three different timeframes of the research.

We hypothesize the following relationships (Fig 1 shows the tested model):

- HP1: higher risk perception, positive outcome expectations and action self-efficacy longitudinally predict the intention to undergo genetic test for BRCA germline mutations.
- HP2: higher health risk attitude and low intolerance to uncertainty have a longitudinal influence on predicted higher intention, planning and action initiation.
- HP3: higher intention and coping self-efficacy longitudinally predict higher planning.
- HP4: higher planning (i.e., action planning, coping planning) and coping self-efficacy longitudinally predict higher action initiation.
- Based on results by Rauscher et al. [14] and Hallowell and colleagues [50], we formulated a research question: (RQ1) is there any difference between personal outcome expectations and family outcome expectations in their association with intention?
- HP5: family-referred narrative gain framed message (vs. self-referred narrative gain framed message) will produce greater intention and action to undergo genetic testing for *BRCA 1/2* germline mutations in at-risk men.

## Method

### Design plan

**Fig 2:**
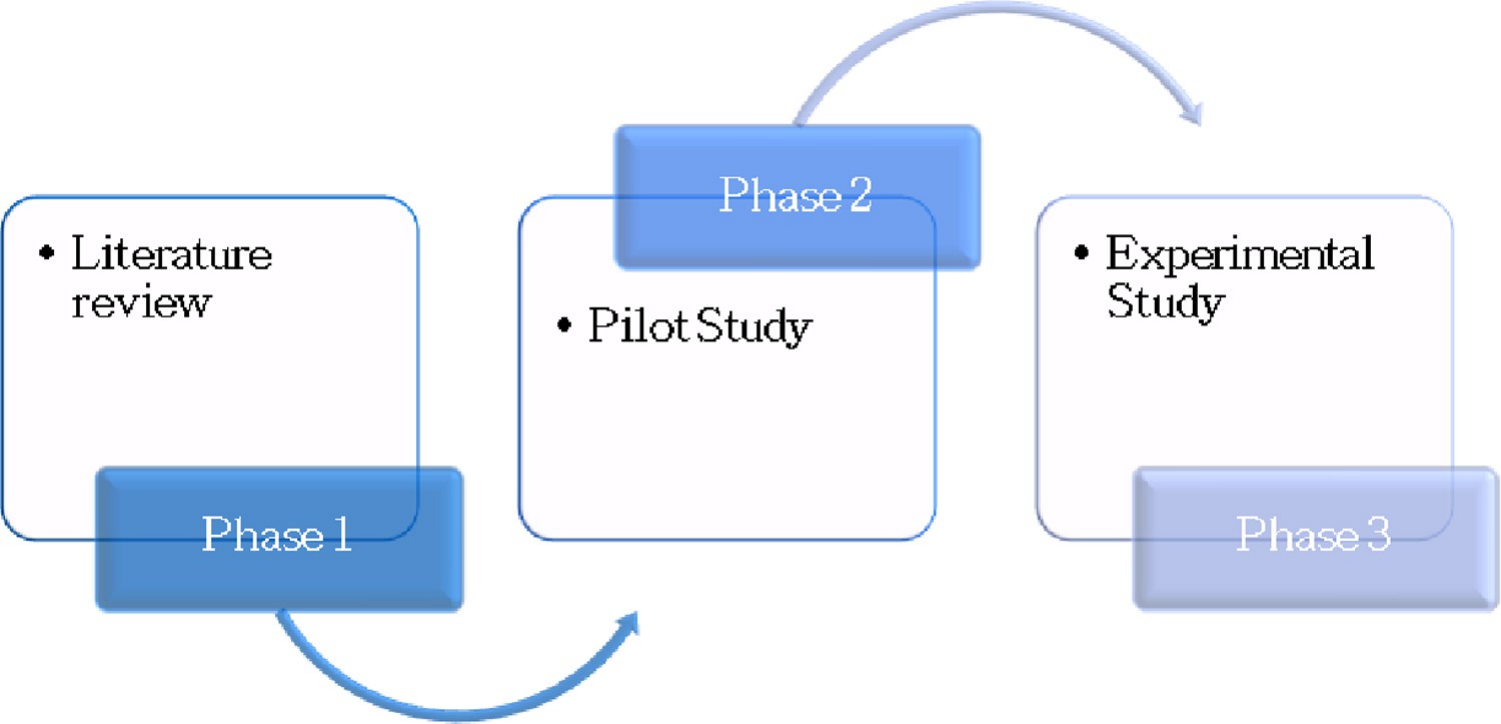
The three phases of the research project.

The research will involve three phases, with results from each phase informing the next phase (see Fig 2). In the first phase, a literature review will be performed to identify other psychological variables influencing individuals’ adherence to evidence-based guidelines on *BRCA1/2* germline genetic testing. Subsequently, a pilot survey will be created in order to test its feasibility and understandability for participants. The third and final phase entails an experimental study analyzing the proposed hypotheses.

### Study plan

The experiment conducted in the third phase will be a Randomized Control Trial with participants receiving one of the two conditions (i.e., self-referred narrative framed message, family-referred narrative framed message) and it tests the main hypotheses and research question of the study. Participants will answer several questions before and after the message/treatment exposure.

### Blinding

Participants will not know the treatment group to which they have been assigned. Personnel who interact directly with the study subjects will not be aware of the assigned treatments (i.e., double-blinded). Personnel who analyze the data collected from the study will be aware of the treatment applied to the given groups; however, they will not interact directly with the participants to ensure double-blindness.

### Sampling plan

According to the registry of the Division of Cancer Prevention and Genetics (IEO), all women with *BRCA1* and/or *BRCA2* germline mutations with at least a male relative will be reached by a phone call and/or an email. During the first contact, a member of the research team informs the female patients about the research purposes and the procedure. The researcher will then ask the women to share the information with their male relative(s) and to invite him (them) to participate in the research. All the information about the research, including an invitation letter, an information sheet, and the consent form, will be also sent by email to the women. If those relatives want to be contacted to participate in the study, a researcher calls them and shares with them the information sheet, the informed consent, and the link to the online survey (via Qualtrics^TM^ Platform). Self-referral of the male participants who have been told by their female relatives about the study will be considered as well. Participants will sign the informed consent before starting to fill the survey and data collection will be done through an online survey via the use of an identifier. Table 1 shows inclusion and exclusion criteria.

**Table 1:** Inclusion and exclusion criteria for the male participants.

Inclusion criteria	Exclusion criteria
Male relatives of patients with an established germline genetic mutation (pathogenic or likely pathogenic variants) of the BRCA1 and / or BRCA2 genes	*BRCA1* and / or *BRCA2* germline genetic screening load detected
Aged ≥ 18	Diagnosis of breast, pancreatic, or prostate cancer
Able to give informed consent	
Able to read, speak, and understand Italian	

Note: ^a^ = this criterion was not present in the study protocol approved by the Ethical Committee.

### Sample size and power calculation

Participants will be 264 males, relatives of women with BRCA1 and/or BRCA2 germline mutations who are patients of the Division of Cancer Prevention and Genetics at the European Institute of Oncology (IEO) in Milan. The IEO is a specialized Hospital and an internationally recognized Cancer Center located in Italy working on research, prevention, diagnosis, and treatment of cancer.

The sample size is determined through an a priori power analysis using GPower 4.0 [51]. Among the imputed parameters it was chosen to include partial η2 = .05, alpha lower than .05, power d (1-B) = .70. Considering that Luszczynska et al. [52] found a η2 = .01, η2 = .05 is a prudential choice. Two groups, corresponding to the two experimental conditions of the study, and 7 covariates were included. The final estimated number of participants is 132, 66 in each group. It should be noted that changing the number of covariates does not change the total number of participants. As of the date of submission of this research plan for preregistration, the data have not yet been collected, created, or realized.

### Randomization

**Fig 3:**
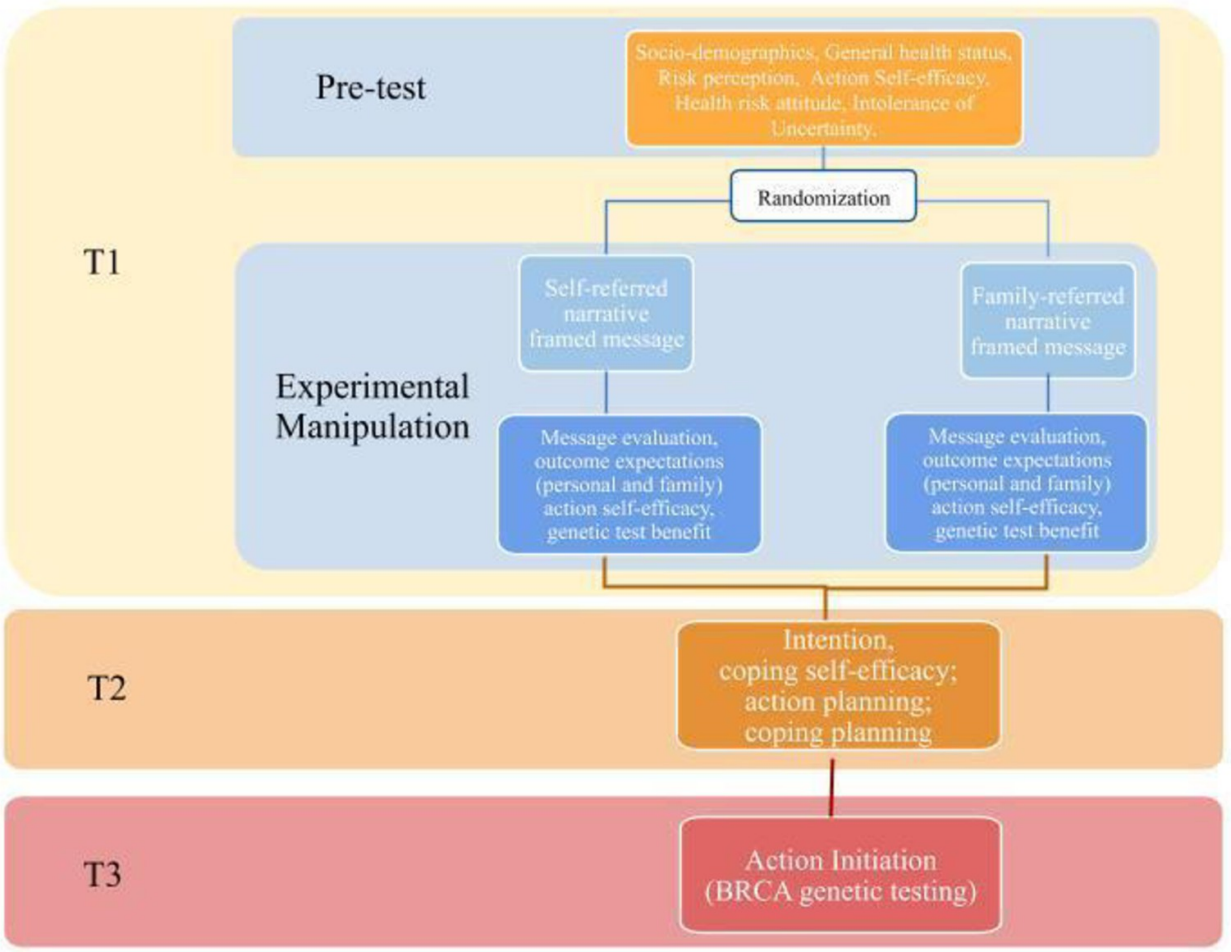
Flowchart of the RCT.

Participants who meet the study inclusion criteria will be randomly assigned to receive one of the two conditions. Fig 3 shows the way that participants will flow through the three assessment points for the study (Time 1, T1; Time 2, T2, Time 3, T3). Randomization will occur during T1, immediately after the data collection of psychological measures. The participants will not be informed of the condition to which they have been allocated.

#### Time 1 assessment

All participants will complete the T1 assessment, via an online survey which will be available for a two-week period. The T1 measures will include demographic, health status, risk perception, health risk aversion, and intolerance of uncertainty. After this evaluation, participants will be randomized and exposed to one message. A manipulation check, the evaluation of the perceived quality of the information presented on the messages, positive outcome expectations, action self-efficacy, benefit for genetic test will be then collected. The constructs of trust in doctors and health literacy included in the original study protocol approved by the Ethical Committee have been not integrated here.

#### Intervention

Participants will be randomly assigned to two groups. Both groups receive a message in which the main character is a man who speaks in first-person. The character has a sister with a *BRCA* germline mutation. After this common introduction, the content of the messages becomes different.

Group 1 receives a self-referred narrative message in which the protagonist explains that he has decided to have a genetic test to discover BRCA germline mutation. He then explains the reasons why this decision is important to himself (e.g., implementing preventive behaviors) and what are the possible benefits for the individual (S1 Fig 1 in S1 File).

Group 2 receives a family-referred narrative message in which the frame is similar to the previous message, but the character this time explains what the benefits for his family are and why his decision to have a genetic test is important to them (S1 Fig 2 in S1 File).

#### Time 2 assessment

The original study protocol approved by the Ethical Committee included a T2 assessment with questions on intention to undergo germline genetic testing and evaluation of the quality of the message content, and a subsequent (3–4 weeks later) T3 with coping self-efficacy, action planning, and coping planning. Since the HAPA model does not state that there should be a waiting time between the measurement of the intention and the coping/action planning, we collapse T2 and T3. Therefore, one-two weeks after the administration of T1, via an online survey, participants reply to questions on intention to undergo germline genetic testing, coping self-efficacy, action planning, and coping planning.

#### Time 3 assessment

Starting from the end of T2 until 3 months later, data on action initiation will be collected (i.e., genetic test for *BRCA1/2* gene germline mutations).

## Measures

### T1 measures

Before the randomization, all participants answer several questions, as follows.

#### Demographic

Self-reported age, education, occupation, degree of relationship with the woman with *BRCA1/2* genes germline mutation, household composition.

#### Health status

Self-reported general health and existing diagnosis for chronic disease will be investigated with a single item each [53]. Response options for general health conditions will be on a 5-point Likert scale. The response options for the item on the existing chronic disease will be binary coded (no—yes, specify).

#### Risk perception

The risk perception will be evaluated through three different measures. Relative risk perception regarding the possibility to develop breast, prostatic, and pancreatic cancer will be investigated with one item each [54, 55]. Response options are on a 7-point Likert scale. The six items of the Health Risk Attitude [56] will be administered to assess how a person would resolve risky health decisions. Response options are on a 7-point Likert scale. The items of the Intolerance of Uncertainty Scale-12 [57] will be applied to measure two dimensions of the intolerance of ambiguity, which are the desire of predictability and uncertainty paralysis. Response options are on a 5-point Likert scale.

### T2 measures

After the message exposure, participants reply to several questions.

#### Manipulation check

Two items created ad hoc evaluate whether participants have read and understood the message content. Multiple-choice answers are used with one correct answer and two incorrect answers as distractors. Participants who fail to answer those questions will be excluded from the analyses.

#### Perceived quality of the message

Three items evaluate whether the message is credible, convincing, and persuasive. Response options are on a 7-point Likert scale.

#### Positive outcome expectation

Eight items will be created ad hoc for this research evaluating positive outcome expectations regarding the participant himself (4 items) and his family members (4 items). Response option will be on a 5-point Likert scale.

#### Action self-efficacy

According to Schwarzer & Luszczynska’s [17] indications, self-efficacy will be assessed through three items as the capability of keeping up with the behavior, by implementing coping strategies. Response option will be on a 5-point Likert scale.

#### Benefit for genetic test

A 5-digit semantic differential will be applied to measure the perceived benefit for genetic tests. Examples of the proposed adjectives are important, relevant, useful, benefic.

#### Intention to undergo genetic testing

The intention of undergoing genetic testing is measured through three items evaluating the urge to engage the behavior. An example of item is: "In the next few months, do you have the intention of planning a genetic screening?". Response options are on a 5-point Likert scale.

#### Coping self-efficacy

Three items created ad hoc for the present research evaluate whether the individual feels to be capable of tackling the possible obstacles and difficulties that could make it difficult to undergo a genetic screening. Response options are on a 5-point Likert scale.

#### Action planning

Three items developed ad hoc for the present research will ask if the participant has planned when, how, and where to undergo the genetic test for BRCA1/2 mutations. Response option will be on a 4-point Likert scale.

#### Coping planning

Four items developed ad hoc for the present research will ask how much the participant thinks to encounter in planning the action. Response option will be on a 5-point Likert scale.

### T3 measures

#### Action

Information on the action initiation (i.e., genetic test for *BRCA1/2* germline mutations) will be collected when the participant takes the appointment for the genetic test and receives the test.

### Primary and secondary outcomes

The primary objective of the present research project is to compare self-referred message to family-referred message promoting men’s adherence to evidence-based guidelines on *BRCA1/2* germline genetic testing. The primary clinical objective is to determine what is the best communication strategy to inform men who are at risk to have a germline mutation because they are relatives of women with such a mutation. The primary hypothesis of the study is that family-referred narrative gain framed message (vs. self-referred narrative gain framed message) will produce greater intention and action to undergo genetic testing for *BRCA 1/2* germline mutations in at-risk men.

The secondary objective is to assess men’s characteristics (demographic information, psychological characteristics, health status) that may explain the intention and the action to undergo genetic test for BRCA germline mutations.

### Ethical considerations

Ethical approval has been acquired from the local ethics committee of the IEO (approval number R1249/20-IEO 1314). Participation is voluntary. All data will be collected in a pseudo-anonymized form. Data will be analyzed in an anonymous form. Data will be treated confidentially and used only by the collaborators in the present study for scientific purposes. No adverse effects are expected for participants; in case of necessity, personal contact of the main researchers will be shared with them.

### Statistical analyses

Data exclusion: The answers to the manipulation check will be analyzed before the main analyses to see whether participants have read the message until the end, understood, and recalled the content.

Missing data: presence of missing data will be checked before conducting the main analyses. If a subject does not complete T1 and T2 that subject will be excluded from the analysis.

Normality of the data will be checked before conducting the main analyses. Non-normality will be handled applying either non-parametric tests or SEM with estimator for non-normal distribution.

Statistical analyses will be performed in SPSS version 25 and the *Lavaan* and *SemPaths* packages in RStudio software.

Preliminary analysis: Pearson’s r correlations among variables.

Main analyses: the hypotheses 1, 2, 3, and 4 will be tested either using structural equation modeling (SEM) or multiple regressions. Research question 1 will be tested either using structural equation modeling (SEM) or ANOVA. In case of non-normality in the data, WLSMV will be applied and the Satorra-Bentler scaled test statistic and robust standard errors were used. In the model specification, the factor loading of the first indicator of each latent variable will be set to 1. The following goodness-of-fit indices will be used to evaluate model-data correspondence: the Chi-square value, the Comparative Fit Index (CFI), and the Root Mean Square Error of Approximation (RMSEA). The model will be considered as acceptable when the CFI is higher than .90 and close to .95 and when the RMSEA is .08 or less. Finally, modification indices and the matrix of standardized correlation residuals will be inspected for potential improvement of model fit. Direct, indirect (or mediate), and total effects were also estimated. To test hypothesis number 5 an ANCOVA will be applied.

- **Hypothesis 1.** Predictors will be: risk perception, positive outcome expectations, action self-efficacy. Covariates: demographic, health status. Outcome variable will be: intention to undergo genetic test for BRCA germline mutations.
- **Hypothesis 2.** Predictors will be: health risk attitude, intolerance to the ambiguity. Covariates: demographic, health status. Outcome variables will be: intention to undergo genetic test for BRCA germline mutations, action and coping planning, action initiation.
- **Hypothesis 3.** Predictors will be: intention to undergo genetic test for BRCA germline mutations, coping self-efficacy. Covariates: demographic, health status. Outcome variables: action and coping planning.
- **Hypothesis 4.** Predictors will be: action and coping planning. Covariates: demographic, health status. Outcome variable: action initiation.
- **Research question 1.** Predictor variable: positive outcome expectations (personal vs family). Outcome: intention to undergo genetic test for BRCA germline mutations.
- **Hypothesis 5.** Predictor variable: treatment/message (family-referred narrative gain framed message vs. self-referred narrative gain framed message). Outcome variables: intention and action to undergo genetic testing for *BRCA 1/2* germline mutations.

## Discussion

The present research intends to evaluate what are the psychological determinants of men’s informed decision-making about genetic tests for *BRCA1/2* germline mutation. Another aim is to test two messages, one focused on the self and one on the family, regarding their capability to inform and persuade men. For these purposes, an experimental study with a longitudinal component will be developed.

Men face *BRCA*-related cancer risks as women do and they may also transmit their mutations to their children. Notwithstanding, men have not received much attention for now. They are under-tested compared to women and the communication is not tailored on their needs [58]. This consideration leads to the need to understand how male relatives of women with *BRCA1/2* germline mutations are motivated to protect themselves and others [13].

The results of the present research provide answers related to the immediate impact of several psychological variables, such as risk perception, positive outcome expectations, self-efficacy have on men’s intention, planning, and action initiation. All those variables are included in the HAPA model which is a well-known theory in the field of health psychology explaining motivational, volitional, and behavioral processes leading to health behavior changes [17, 18, 59]. The study will also analyze the role of health risk aversion and intolerance of ambiguity, which are variables linked to decision-making in health domains [31–34, 37, 60]. Finally, the research will inform regarding the effectiveness of two messages tailored on the specific men’s needs and focused one on the self and one on the family.

Data gathered from this study may inform health care providers, policy makers, and public health managers about the psychological variables implicated in the decision-making and the best communication strategies considering the various stages of changes, health risk aversion, and intolerance to the ambiguity. The study will give knowledge on how to modulate messages for the male population aimed at informing their decision-making and will guide genetic counsellors [61] for future interventions.

A good communication strategy may induce men to overcome their fear of stigmatization and to seek for information, since one of the most important barriers for men is the lack of knowledge about *BRCA1/2* germline mutations and their consequences. Moreover, it may also induce them to protect themselves and other family members by asking for genetic counseling. The present research tested two gain framed narrative messages written in the first-person vs. third person/family. Therefore, results from the present research will make it possible to adapt the communication of personal and psychological characteristics of the individuals, to inform their decision-making. The more individuals are correctly informed, the more they are free to choose between being tested or not for *BRCA1/2* germline mutations, the more the ethical balance between the benefits and risks of the genetic test will be reduced.

## Supporting information

S1 File (DOCX)

## Abbreviations

BRCA1: BReast CAncer 1 gene
BRCA2: BReast CAncer 2 gene
HAPA: Health Action Process Approach
HBOC: Hereditary Breast and Ovarian Cancer Syndrome
IEO: European Institute of Oncology
IU: Intolerance of Uncertainty
WLSMV: weighted least square mean and variance adjusted
