# Supplementary material for: A randomized controlled trial comparing self-referred message to family-referred message promoting men’s adherence to evidence-based guidelines on BRCA1/2 germline genetic testing: A registered study protocol
Source: PLoS One. 2022 Apr 8;17(4):e0266327. doi: 10.1371/journal.pone.0266327 (PMC8992988; doi:10.1371/journal.pone.0266327)

Supporting Information 1

Figure 1. Narrative gain framed message with content focusing on family members.


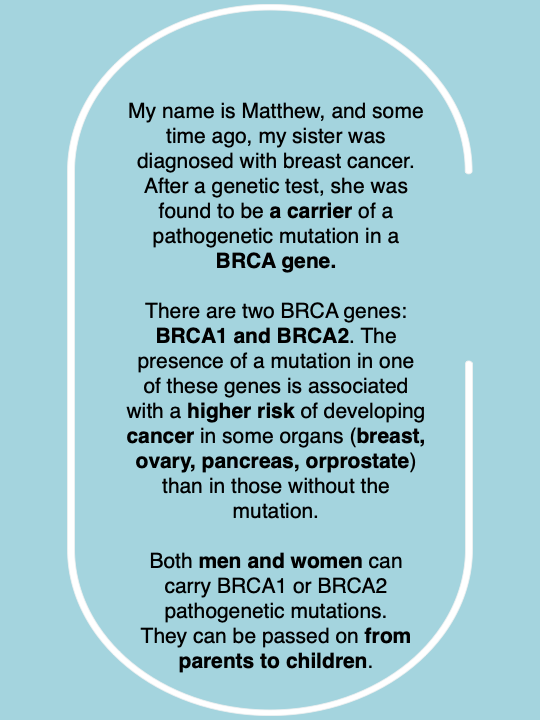

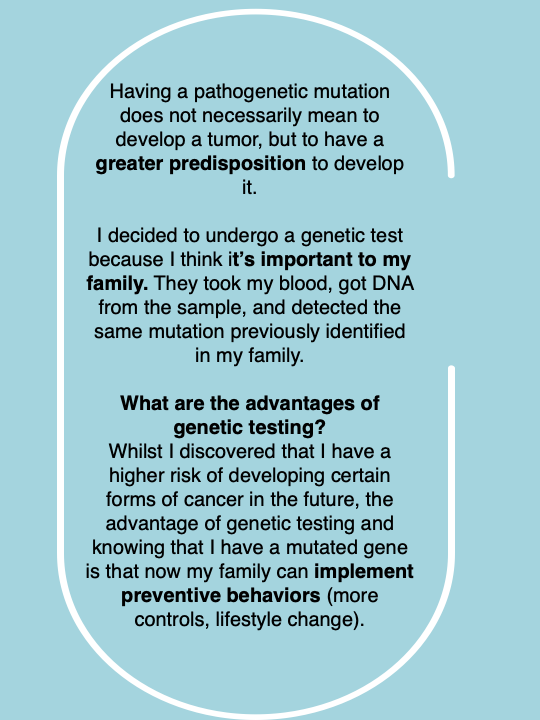


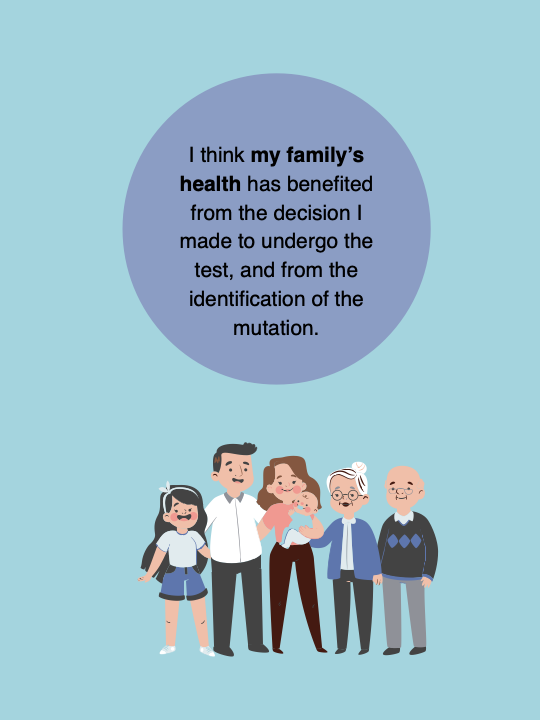

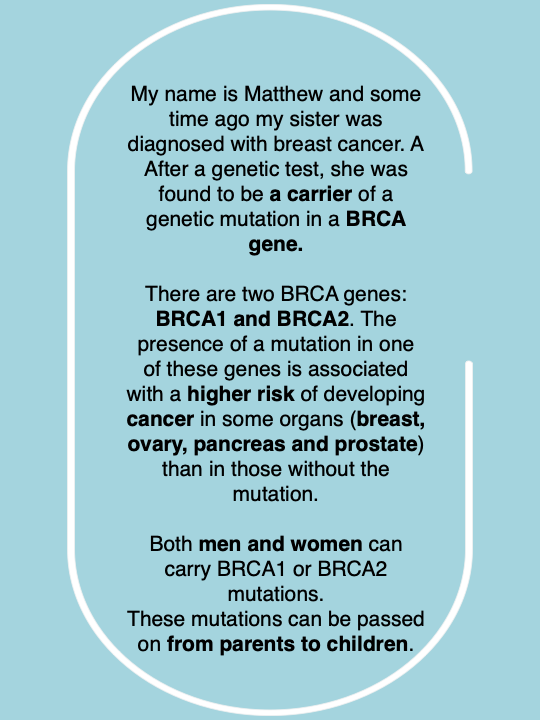


Figure 2. Narrative gain framed message with content focusing on the individual.


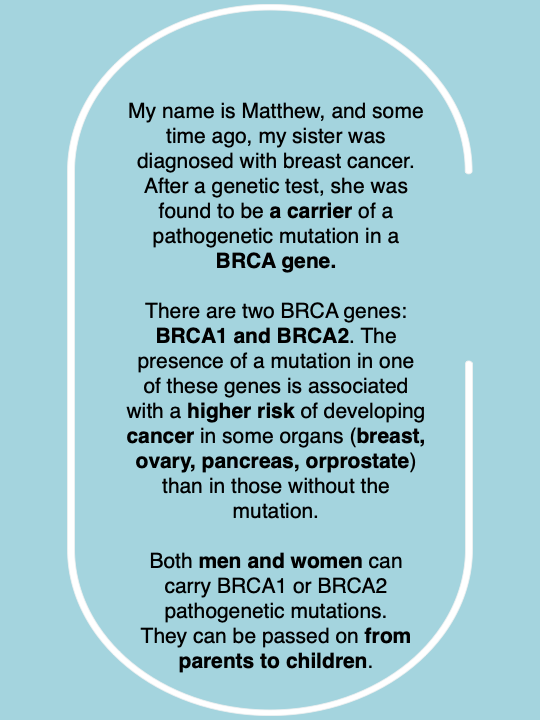

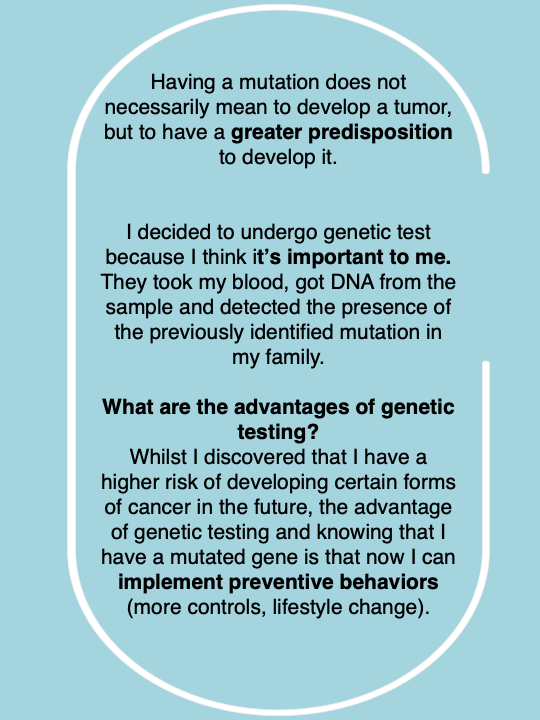


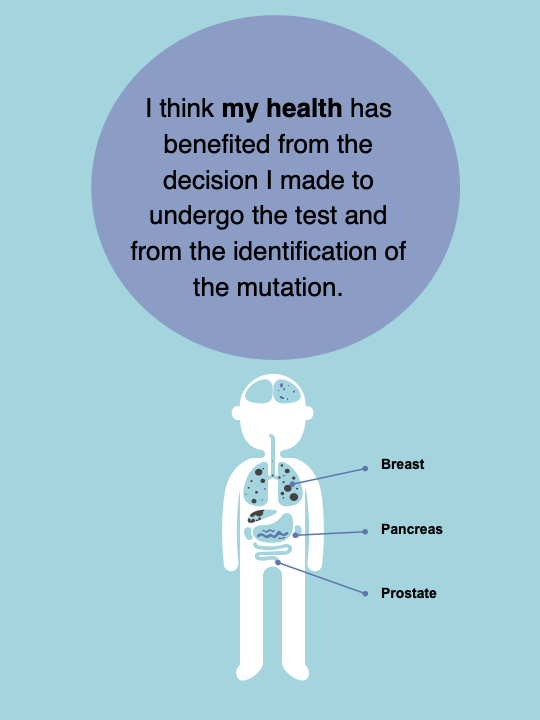

Supplement: S1 File — (DOCX) [file pone.0266327.s001.docx]
